# Assessment of lip morphology changes after bimaxillary orthognathic surgery using stereophotogrammetry

**DOI:** 10.1590/0103-644020256543

**Published:** 2026-01-09

**Authors:** Juliana Rodrigues Rozatto, Lucas Moura Sousa, Ana Maria Bettoni Rodrigues, Marco Antonio M. Rodrigues da Silva, Alexandre Elias Trivellato, Cássio Edvard Sverzut

**Affiliations:** 1 Department of Oral & Maxillofacial Surgery, and Periodontology, University of São Paulo (USP), Ribeirão Preto School of Dentistry(FORP), Ribeirão Preto, SP, Brazil; 2 Department of Restorative Dentistry, University of São Paulo (USP), Ribeirão Preto School of Dentistry(FORP), Ribeirão Preto, SP, Brazil

**Keywords:** Stereophotogrammetry, Three-dimensional image, Orthognathic surgery, Lip measurements

## Abstract

This study aimed to evaluate changes in upper and lower lip morphology after bimaxillary orthognathic surgery using stereophotogrammetry at the preoperative (T0) and postoperative periods of 6 months (T1) and 1 year (T2). For this purpose, 3D images of 11 patients (9 women and 2 men), with an average age of 33.5 years, who underwent surgery in hospitals associated with the Residency Training Program, School of Dentistry of Ribeirão Preto, University of São Paulo, from 2013 to 2019, were analyzed. The software Vectra M3 was used to calculate linear measurements, distance from lip surfaces, and areas. A significant decrease in the lower lip height (p = 0.0621), surface (p = 0.0435), and area (p = 0.0042), as well as an increase in upper lip area (p = 0.029), were observed from T0 to T1. All measurements remained stable from T1 to T2. Additionally, the ages of patients were correlated with the variables at T0, T1, and T2, and the data showed a strong correlation with height and distance of the lower lip vermilion, as well as lip width, particularly at T2. In conclusion, stereophotogrammetry proved to be a valuable tool for assessing soft tissue changes, revealing a significant increase in upper lip area and a decrease in lower lip area, as well as height, and distance from its surface.

## Resumo

Este estudo teve como objetivo avaliar as mudanças na morfologia dos lábios superior e inferior após cirurgia ortognática bimaxilar, utilizando estereofotogrametria nos períodos pré-operatório (T0) e pós-operatórios de 6 meses (T1) e 1 ano (T2). Para isso, foram analisadas imagens 3D de 11 pacientes (9 mulheres e 2 homens), com idade média de 33,5 anos, que foram submetidos à cirurgia em hospitais associados ao Programa de Residência em Cirurgia e Traumatologia Bucomaxilofacial da Faculdade de Odontologia de Ribeirão Preto, Universidade de São Paulo, entre 2013 e 2019. O software Vectra M3 foi utilizado para calcular medidas lineares, distâncias das superfícies labiais e áreas. Observou-se uma redução significativa na altura do lábio inferior (p = 0,0621), na sua superfície (p = 0,0435) e na área (p = 0,0042), assim como um aumento na área do lábio superior (p = 0,029) do T0 para o T1. Todas as medidas permaneceram estáveis do T1 para o T2. Além disso, as idades dos pacientes foram correlacionadas com as variáveis nos tempos T0, T1 e T2, mostrando forte correlação com a altura e distância do vermelhão do lábio inferior, assim como com a largura do lábio, especialmente no T2. Em conclusão, a estereofotogrametria demonstrou ser uma ferramenta valiosa para a avaliação das alterações dos tecidos moles, revelando um aumento significativo na área do lábio superior e uma redução na área, altura e distância da superfície do lábio inferior.

**Figure:**
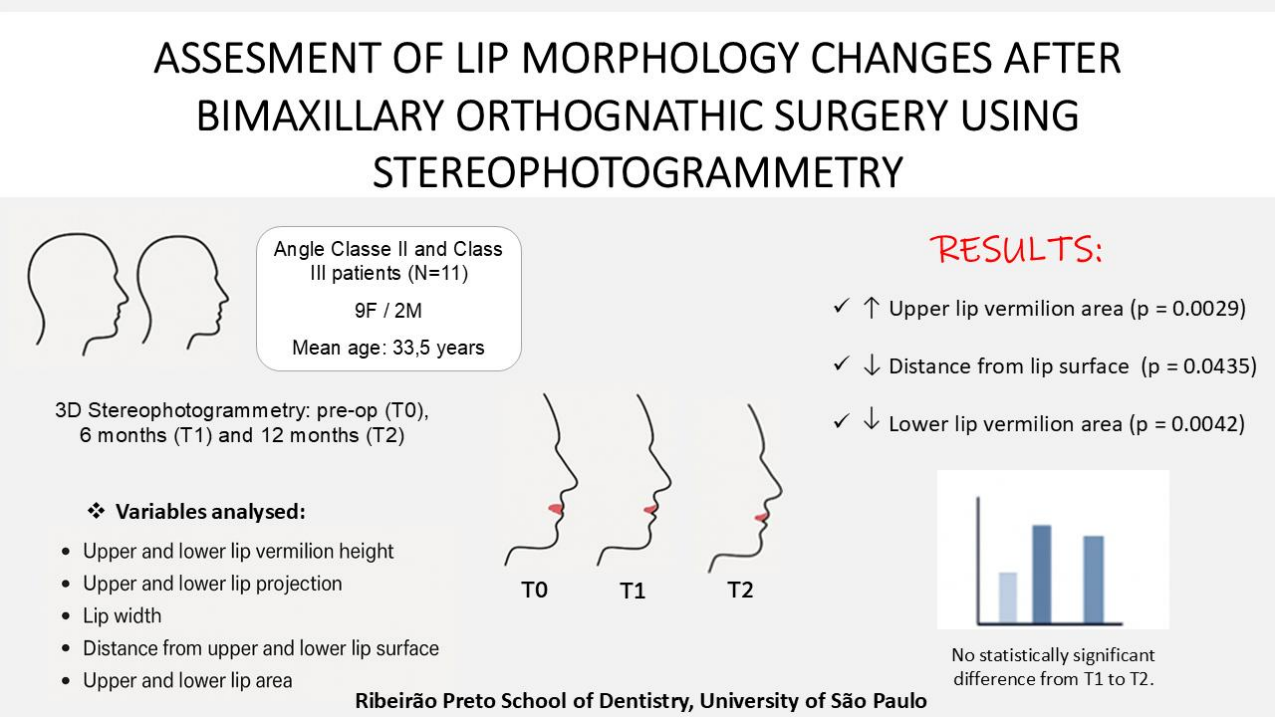

## Introduction

Orthognathic surgery aims to improve the stomatognathic system function and facial symmetry and harmony^1^ ^,^ ^2^. Appearance is a primary concern for most people seeking this surgical treatment, and the lips are essential components of facial aesthetics. The three-dimensional repositioning of bones during orthognathic surgery can lead to varying degrees of significant changes in the patient's face, such as alterations to the nose, lips, zygomatic regions, and submandibular area. However, predicting the effect of orthognathic surgery on the soft tissue profile is not straightforward^1^ ^,^ ^3^.

In recent years, technology has provided new noninvasive tools for facial morphology analysis, with stereophotogrammetry standing out as a method with proven accuracy and repeatability^4^ ^,^ ^5^ ^,^ ^6^. This technology enables fast, accurate, and safe three-dimensional imaging of soft tissue while also providing additional information such as surface color and texture. Consequently, it is possible to obtain a better surface image with higher resolution and, therefore, improved quality^4^ ^,^ ^7^ ^,^ ^8^.

Due to the importance of lip symmetry and projection on facial appearance, it is crucial to assess changes in lip morphology prior to orthognathic surgery accurately. This helps optimize surgical planning and facilitates communication between the surgeon and patient, which is essential for achieving a satisfactory final result^7^ ^,^ ^9^ ^,^ ^10^. Therefore, the aim of the present study was to quantitatively evaluate upper and lower lip morphology changes in patients undergoing two-jaw orthognathic surgery using stereophotogrammetry.

## Materials and methods

This project was analyzed and approved by the Research Ethics Committee of the School of Dentistry of Ribeirão Preto, University of São Paulo - USP (CAAE 16663919.2.0000.5419). All patients received the research information through an informed consent form.

The inclusion criteria consisted of skeletally mature patients undergoing bimaxillary orthognathic surgery. The surgical techniques consisted of a Le Fort I osteotomy, performed according to the technique proposed by Bell in 1975^11^, and a bilateral sagittal split osteotomy of the mandibular ramus, modified by Epker in 1977^12^. The internal fixation systems used were as follows: for the maxilla, a 1.5 mm conventional system was applied, consisting of four plates fixed with four screws with 5.0 mm in length each, and, for the mandible, a 2.0 mm conventional system was applied, consisting of one plate fixed with four screws with 5.0 mm in length on each side. Additionally, patients who obtained 3D images with neutral facial expression in the preoperative and at 6 and 12 months after surgery.

Conversely, the exclusion criteria were if the patients underwent any other surgical procedure during transoperative or postoperative period (such as mentoplasty, segmental surgery, dental implants, bone reconstruction, among others), presented with congenital anomalies (e.g., cleft lip and/or palate, syndromes), had a history of craniofacial trauma, underwent aesthetic procedure in the perioral region (e.g., rhinoplasty, botulinum toxin application, lip filling with hyaluronic acid), required wound suturing techniques other than continuous “U” sutures (e.g., V-Y suture), experienced any postoperative complication (e.g., infection, fixation system failure), or failed to have preoperative or postoperative photographs taken at 6 months and 1 year. In addition, movements greater than 10 mm in anteroposterior direction and 8 mm of clockwise or counterclockwise rotation were not included in the study.

### Image Acquisition

The images were obtained in the Laboratory for Research into Electromyography of the Stomatognathic System (LAPESE), rigorously observing the protocols established by said laboratory. Three images were obtained from each patient: one during the preoperative period (T0), one at 6 months postoperatively (T1), and one at 1 year postoperatively (T2). The photos were captured using the Vectra® M3 system (Canfield Scientific, Fairfield, NJ, USA), which has a geometric resolution of 1.2 mm (triangle edge length). This system consists of a ground support composed of 3 modules, featuring 2 cameras and 2 intelligent flash units each that capture two-dimensional images at different angles, thus allowing the formation of a 3D image. The entire image capture process occurs within 3.5 milliseconds, making the system immune to the movement of the patient. Also, the analyses were performed by the same operator (JRR).

**Figure 1:**
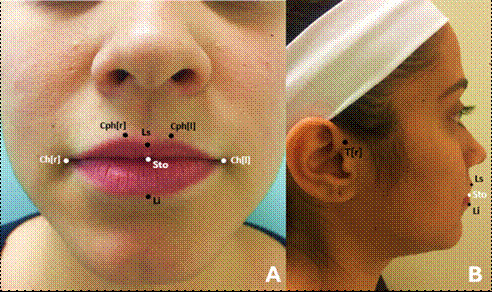
(A) Front and (B) side view of the 8 landmarks used in the study. Black dots are marked on the face, while white dots are directly marked in the software after image capturing.

To perform these analyses, 8 landmarks were selected, as proposed by Farkas ^8^ (Table 1 and Figure 1). Firstly, Labiale Superius (Ls), right Crista Philtri (Cph[r]), left Crista Philtri (Cph[l]), Labiale Inferius (Li), and right Tragion (T[r]) landmarkers were manually performed on the face with the aid of a marker (Sharpie, Tennessee, USA). After markings, patients were instructed to sit on a height-adjustable seat and positioned in front of the central module of the equipment (approximately 112 cm), looking directly at a mirror, strategically located below the central module. In addition, the patient's positioning was finalized using horizontal and vertical lines provided by the program and viewed in real time on a flat screen monitor (Dell Technologies Inc., Round Rock, Texas, USA). The images were captured only when the patient's positioning was in accordance with these guidelines, and with spontaneous facial expression, lips at rest, and teeth in occlusion. Also, right Cheilion (Ch[r]), left Cheilion (Ch[l]), and Stomion (Sto) landmarkers were directly marked in the software VECTRA® Analysis Module (VAM), as they are difficult to access points on the face and are anatomically well delimited in images.

**Table:** 1. Landmarks used to perform analyses in this study, with their abbreviations and definitions.

Abbreviation	Landmark	Definition
Ls	Labiale Superius	Midline at the beginning of the upper lip vermilion
Cph[r], Cph[l]	Crista Philtri	At each raised edge of the filter above the upper vermilion line
Ch[r] Ch[l]	Cheilion	Lip Commissure
Sto	Stomion	Intercession of the facial midline and the horizontal cleft lip
Li	Labiale Inferius	Midline at the beginning of the lower lip vermilion
T[r]	Tragion	Upper margin of the tragus

### Selection of measurements (linear, surface, angle, and area measurements)

The following linear measurements were evaluated: upper lip height (Ls-Sto), lower lip height (Sto-Li), upper lip projection (T[r]-Ls), lower lip projection (T[r]-Li), and lip width (Ch[r]-Ch[l]). To calculate the distance from the lip surface, Ls-Sto for the upper lip and Sto-Li for the lower lip; however, a curved line surrounding the vermilion was used instead of a straight line. The lip area was assessed by outlining the vermilion using specific landmarkers. For the upper lip, the outline included Ch[r]-Cph[r]-Ls-Cph[l]-Ch[l]-Sto-Ch[r], while for the lower lip, it included Ch[r]-Sto-Ch[l]-Li-Ch[r].

### Reliability degree

To test the reliability of landmarks positioning on the face, two tests were previously performed using hand marking. The first test was an intra-operator test, in which the same 9 hand-marked points were identified in 10 randomly selected participants (5 women and 5 men) with a 1-week interval. After digitalizing the 12 points, the exact measurements used in the study were calculated for both periods and compared using the Intra-Class Correlation Coefficient (ICC). The reproducibility of the markings was considered excellent, with a score of at least 86%.

The second test was an inter-operator test, which consisted of one experienced and calibrated operator and two uncalibrated operators. The sample consisted of 10 participants, all women. After performing the same markings for each participant, comparisons between operators were made using Principal Component Analysis (PCA).

### Statistical analysis

In this study, the primary outcome was to evaluate the changes in facial measurements of patients undergoing orthognathic surgery over time, specifically comparing preoperative values (T0) with 6 months (T1) and 1 year (T2) postoperative measurements. Although the variables showed a normal distribution according to the Kolmogorov-Smirnov test (p ≥ 0.05), a non-parametric analysis was chosen due to the small sample size (n = 11). The Friedman test, followed by Dunn’s multiple comparison test, was performed. Finally, the Spearman Correlation Test (ranging from -1 to 1) was performed to compare age with variables.

## Results

This is a retrospective study in which 92 medical records of patients who underwent orthognathic surgery from August 2013 to August 2019 at the Residency Training Program, School of Dentistry of Ribeirão Preto, University of São Paulo, were analyzed. According to the inclusion and exclusion criteria, 11 medical records were selected, 9 from women (81.1%) and 2 from men (18.9%) aged 21 to 55 years (mean age 33.5 years). In this sample, only two patients were over 40 years old (specifically, 42 and 55 years old). The study population consisted predominantly of White patients (10/11), with a single Black patient (1/11). Of these, 3 (27.2%) patients were preoperatively diagnosed as Angle Class III and 8 (72.7%) with Class II. The mean movements proposed in the preoperative surgery planning for the maxilla were: 4.09 mm (± 1.94) anteroposterior advancement, 3 mm (± 3.68) of anterior intrusion, and 2.13 mm (± 3.27) of posterior intrusion. For the mandible, these measures were: 2.98 mm (± 3.2) of anteroposterior advancement and 3.5 mm (± 3.7) of counterclockwise rotation.

In total, 33 photos were evaluated at VAM. The PCA results showed a divergence of less than 10% from the average value obtained between the operators. The mean values ​​and standard deviation of variables at times T0, T1, and T2 are described in Table 2. There was no statistically significant difference between periods when analyzing the following measurements: upper (Ls-Sto) and lower (Sto-Li) vermillion height (p = 0.6293 and p = 0.0621, respectively), upper (T[r]-Ls) and lower (T[r]-Li) projection (p = 0.4698 and p = 0.8438, respectively), lip width (Ch[r]-Ch[l]) (p = 0.0927), and distance from upper lip surface (Ls-Sto) (p = 0.4026).

**Table 2:** Mean ± standard deviation and 95% confidence interval of facial measurements obtained at the preoperative period (T0), and at 6 months (T1) and 1 year (T2) postoperatively.

Linear measurements (mm)	T0	T1	T2	p-value
Upper lip vermilion height (Ls-Sto)	7.6 ±1.28 (95% CI: 5.93 - 8.83)	8.0 ±1.3 (95% CI: 6.68 - 9.84)	8.0 ±1.4 (95% CI: 6.80 - 9.80)	0.6293
Lower lip vermilion height (Sto-Li)	10.3 ±1.3 (95% CI: 7.65 - 12.87)	9.2 ±0.9 (95% CI: 7.59 - 10.98)	9.3 ±0.8 (95% CI: 6.42 - 11.54)	0.0621
Lip width (Ch[r]-Ch[l])	50.2 ±2.9 (95% CI: 45.82 - 53.19)	51.1 ±2.5 (95% CI: 48.34 - 54.31)	50.1 ±3.5 (95% CI: 46.41 - 53.19)	0.0927
Upper lip projection (T[r]-Ls)	122.4 ±4.7 (95% CI: 114.5 - 126.5)	123.7 ±6.6 (95% CI: 115.7 - 131.6)	124.1 ±6.2 (95% CI: 117.9 - 130.5)	0.4698
Lower lip projection (T[r]-Li)	127.7 ±6.2 (95% CI: 120.2 - 135.9)	127.3 ±7.3 (95% CI: 117.3 - 136.0)	127.7 ±6.8 (95% CI: 118.9 - 134.9)	0.8438
Surface Measurements (mm)				
Distance from the upper lip vermilion surface (Ls-Sto)	7.9 ±1.3 (95% CI: 6.11 - 8.99)	8.2 ±1.7 (95% CI: 6.69 - 10.21)	8.3 ±1.5 (95% CI: 6.96 - 10.56)	0.4026
Distance from the lower lip vermilion surface (Sto-Li)	11.1 ±2.7 (95% CI: 7.83 - 14.28)	9.7 ±1.9 (95% CI: 7.77 - 11.80)	9.7 ±2.3 (95% CI: 6.53 - 12.13)	0.0435*
Area measurements (cm²)				
Upper lip vermilion area (Ch[r]-Cph[r]-Ls-Cph[l]-Ch[l]-Sto-Ch[r])	3.3 ±0.8 (95% CI: 2.58 - 4.14)	3.6 ±0.8 (95% CI: 2.91 - 4.90)	3.6 ±0.9 (95% CI: 2.72 - 4.69)	0.0029**
Lower lip vermilion area (Ch[r]-Sto-Ch[l]-Li-Ch[r])	4.2 ±1.32 (95% CI: 2.65 - 5.50)	3.7 ±0.9 (95% CI: 2.93 - 4.56)	3.5 ±0.8 (95% CI: 2.56 - 4.42)	0.0042***

* Represents statistical difference observed by the Friedman Test (p< 0.05). Post hoc Dunn’s test revealed a difference in the distance from the *lower lip vermilion surface* between T0 and T2 (p = 0.0315); in the *upper lip vermilion area* between T0 and T1 (p = 0.0167) and T0 and T2 (p = 0.0085); and in the *lower lip vermilion area* between T0 and T2 (p = 0.0042).

**Figure 2:**
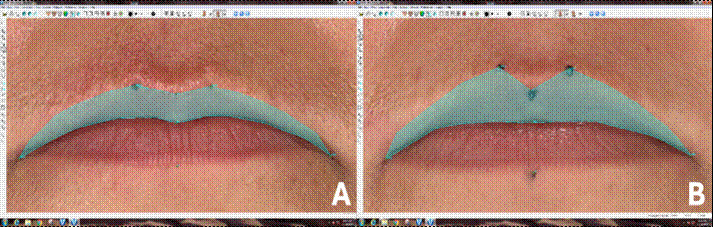
3D photo of the upper lip of the same patient in the preoperative **(A)** and postoperative periods of 6 months (B) after two-jaw orthognathic surgery, showing remarkable changes in the height (Li-Sto) and area (Ch[r]-Cp [r -Ls-Cph[l]-Ch[l]-Sto-Ch[r]) of upper lip (area delimited in green).

**Figure 3:**
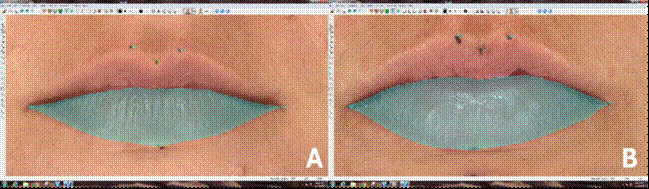
3D photo of the upper lip of the same patient in the preoperative **(A)** and postoperative periods of 6 months (B) after two-jaw orthognathic surgery, showing remarkable changes in the height (Li-Sto) and area (Ch[r]-Sto-Ch[l]-Li-Ch[r]) of the lower lip (area delimited in green).

However, differences were observed for upper lip area (Ch[r]-Cph[r]-Ls-Cph[l]-Ch[l]-Sto-Ch [r]) (p = 0.0029), both between T0 and T1 (p = 0.0167) and T0 and T2 (p = 0.0085). A decrease in distance from lip surface (Sto-Li) (p = 0.0435) between T0 and T2 (p = 0.0315). Also, reduction in the lower lip vermilion area (Ch[r]-Sto-Ch[l])-Li-Ch[r]) (p = 0.0042) was observed, specially between T0 and T2 (p = 0.0042) of the lower lip was observed from T0 to T1 (Figures 2 and 3). However, in none of the cases was there a statistically significant difference from T1 to T2.

Spearman's correlation showed substantial and statistically significant relationships between age and the height and distance of the lower lip vermilion, as well as lip width, particularly at T2 (Table 3). The longer the postoperative time, the lower the height, the distance from the lower lip surface, and the vermilion area between T1 and T2.

**Table 3:** Correlation between variables and periods with the age of patients using Spearman's Correlation Test.

Linear measurements (mm)	T	**Spearman *r***	95% CI	*p-value*
Height of the upper lip vermillion	T0	-0.07306	-0,6565 to 0,5651	0.8325
Height of the upper lip vermillion	T1	-0.2466	-0,7466 to 0,4314	0.4619
Height of the upper lip vermillion	T2	-0.4795	-0,8442 to 0,1889	0.1376
Height of the lower lip vermillion	T0	-0.3196	-0,7797 to 0,3646	0.3352
Height of the lower lip vermillion	T1	-0.5388	-0,8658 to 0,1105	0.0908
Height of the lower lip vermillion	T2	-0.7169	-0,9238 to -0,1856	0.0161*
Lip width	T0	0.5114	-0,1477 to 0,8560	0.1107
Lip width	T1	0.5616	-0,07804 to 0,8737	0.0761
Lip width	T2	0.7032	0,1588 to 0,9197	0.0191*
Upper lip projection	T0	-0.2374	-0,7423 to 0,4393	0.4788
Upper lip projection	T1	-0.3333	-0,7857 to 0,3512	0.3137
Upper lip projection	T2	0.009133	-0,6071 to 0,6185	0.9839
Lower lip projection	T0	-0.1918	-0,7200 to 0,4771	0.5698
Lower lip projection	T1	-0.2968	-0,7696 to 0,3863	0.3723
Lower lip projection	T2	-0.08219	-0,6617 to 0,5588	0.8117
Surface measurements (mm)				
Distance from the upper lip vermillion	T0	-0.1187	-0,6819 to 0,5329	0.7278
Distance from the upper lip vermillion	T1	-0.3516	-0,7935 to 0,3330	0.2868
Distance from the upper lip vermillion	T2	-0.4703	-0,8408 to 0,2002	0.1458
Distance from the lower lip vermillion	T0	-0.242	-0,7444 to 0,4354	0.4701
Distance from the lower lip vermillion	T1	-0.5069	-0,8543 to 0,1537	0.1143
Distance from the lower lip vermillion	T2	-0.7071	-0,9209 to -0,1664	0.0179*
Area measurements (cm²)				
Upper lip vermillion area	T0	0.08219	-0,5588 to 0,6617	0.8117
Upper lip vermillion area	T1	-0.06849	-0,6539 to 0,5682	0.8433
Upper lip vermillion area	T2	-0.1644	-0,7061 to 0,4987	0.6274
Lower lip vermillion area	T0	-0.379	-0,8049 to 0,3046	0.2488
Lower lip vermillion area	T1	-0.5616	-0,8737 to 0,07804	0.0761
Lower lip vermillion area	T2	-0.4429	-0,8304 to 0,2332	0.173

* Represents statistical difference (p< 0.05).

## Discussion

In this study, changes in lip vermilion morphology before and after two-jaw orthognathic surgery were evaluated, since changes in this region are one of the main concerns of patients because they are directly related to facial aesthetics. In general, changes were observed over time in the patients' lips after orthognathic surgery^19^. Notably, there was an increase in the upper lip vermilion area (p = 0.0029) and upper lip vermilion height (p = 0.6293), and a decrease in the lower lip vermilion area (p = 0.0042) and lower lip vermilion height (p = 0.0621).

The evaluation of facial changes after orthognathic surgery has been a topic of interest since the early 1970s^13^. Currently, several methods have been proposed to evaluate these changes, including magnetic resonance imaging, ultrasonography, cone-beam computed tomography, and laser scanning^4^ ^,^ ^7^ ^,^ ^8^ ^,^ ^14^. While these methods provide 3D images, they have certain limitations, such as high cost, significant exposure to ionizing radiation, poor resolution, and the inability to capture skin color and texture^8^ ^,^ ^14^ ^,^ ^15^ ^,^ ^16^ ^,^ ^17^. The stereophotogrammetry methods stand out as they can capture three-dimensional images quickly, accurately, and non-invasively, providing high-resolution images and enabling studies with high accuracy^18^.

As aforementioned, the surgical procedures to reposition the facial skeleton can lead to varying degrees of changes in the patient's face, especially in the lip. Data from a systematic review demonstrated a relationship between soft and hard tissue movements in patients undergoing orthognathic surgery^20^. According to these authors, a direct relationship was found between the upper lip and upper incisors, ranging from 36 to 100% across different studies. They attributed these variations to factors such as the magnitude of maxillary advancement, the surgical technique employed, and the different types of sutures used.

The upper lip vermilion height and the distance from its surface showed no significant differences from T0 to T1. However, a significant increase in area was observed, indicating greater exposure of the lip in lateral regions while maintaining height in the central region. Kim et al.^19^ evaluated Class III patients who underwent maxillary advancement and mandibular retrusion through CT scan superimpositions and similarly observed more pronounced changes in the lateral regions of the upper lip compared to the central area. According to these authors, this was likely due to mandibular retrusion movement and laxity of soft tissues near the midline of the face.

Altug-Atac et al. (2008 apud Olate S. et al, 2016, p.7)^20^ observed that the significant aesthetic change caused by bimaxillary surgery was mainly due to the new position of the lower lip. Additionally, Lu et al. (2003 apud Olate S. et al, 2016, p.7)^20^ stated that the lower lip is influenced not only by the position of the upper and lower incisors but also by perioral muscles. Therefore, changes in overjet lead to changes in lower lip position. It is also important to highlight that the movement of the maxilla can be correlated with changes in lip volume^19^.

In this current study, the height, distance from the lip surface, and lower lip vermillion area decreased from T0 to T1, similar to results obtained by Almukhtar et al.^21^, who observed lower lip thinning after bimaxillary surgery. The authors evaluated these changes using color maps obtained by superimposition of cone-beam computed tomography. However, this evaluation method was reported as a descriptive analysis method, which does not provide accurate and reliable measurements, such as stereophotogrammetry.

Likewise, the data presented by Vivas-Castilho et al.^22^ demonstrated that orthognathic surgery in patients with retrusive profiles can statistically increase the upper lip concavity angle, vermilion length, and upper lip’s sagittal distance to the Barcelona line (a tangent line passing through the nasion and perpendicular to the natural orientation of the head). The findings were interpreted as indicating a more projected and everted upper vermilion.

No statistical difference was found in lip width, unlike the results found by Gerbino et al.^23^, who described an enlargement between labial commissures after maxillo-mandibular advancement. A possible explanation for this discrepancy is that Gerbino et al.^23^ used 3D laser scanning in their analysis, which may be more sensitive to subtle soft tissue changes. Differences in imaging techniques, resolution, and landmark identification can affect the accuracy and precision of measurements, potentially leading to variations in observed outcomes.

The correlation analysis (Table 3) showed that before surgery (T0), no significant relationship was found between the variables and the patients’ age. However, after 1 year (T2), age inversely influenced changes in some variables in a statistically significant way. Postoperative facial changes after orthognathic surgery are influenced by multiple factors, including the magnitude and type of skeletal movements, pre-existing asymmetries, sex, age, soft tissue thickness and elasticity, surgical technique and fixation method, as well as postoperative care and rehabilitation ^21^ ^,^ ^23^. Age cannot be considered as an isolated factor in determining postoperative changes following orthognathic surgery, as many variables can influence these outcomes. However, our study demonstrated that age may be associated with changes in certain variables, particularly after one year, such as the height and distance of the lower lip vermilion, as well as lip width.

The results showed that, with increased age, there was a decrease in vermilion height, distance from the lip surface, and lower lip area. The lower third of the face, particularly in the mandible region, has thicker soft tissue that includes the mimetic and masticatory muscles. As age progresses, these muscles lose tonicity^24^, which may cause lower lip changes.

Also, it is important to highlight that facial edema decreases rapidly in the first 3 postoperative weeks, and a significant decrease continues to occur between 6 months and 1 year after surgery^25^. In our study, no statistically significant differences between 6 months and 1 year were observed, suggesting that by 6 months, lips would have reached their definitive morphology.

A limitation of this study is the small sample size, which resulted from the strict inclusion and exclusion criteria. Further subdividing the population based on the magnitude or direction of jaw movement or gender would have created even smaller subgroups, limiting the applicability of statistical analyses. Based on our data for upper lip vermilion height (Ls-Sto), with a mean difference of 0.4 mm and a standard deviation of 1.3, a sample size of 85 patients would be required to achieve 80% statistical power for detecting this difference in a paired analysis (α = 0.05). Our current study included only 11 patients, indicating that it is underpowered to detect such minor differences. Nevertheless, the findings provide valuable preliminary insights and can inform the design of future studies with larger cohorts.

Although the only change observed in the upper lip was an increase in vermilion area, the lower lip showed a decrease in this area, as well as reductions in height and distance from its surface. In this context, bimaxillary orthognathic surgery results in changes in lip vermilion morphology. Age was not an isolated determinant but was associated with specific changes after one year, particularly in lower lip morphology. No significant differences were observed between 6 months and 1 year postoperatively, suggesting that the lips largely reach their final shape by 6 months.
